# Mathematical Models in the Description of Pregnane X Receptor (PXR)-Regulated Cytochrome P450 Enzyme Induction

**DOI:** 10.3390/ijms19061785

**Published:** 2018-06-15

**Authors:** Jurjen Duintjer Tebbens, Malek Azar, Elfriede Friedmann, Martin Lanzendörfer, Petr Pávek

**Affiliations:** 1 Department of Biophysics and Physical Chemistry, Faculty of Pharmacy, Charles University, Heyrovského 1203, 500 05 Hradec Kralove, Czech Republic; azarm@faf.cuni.cz; 2 Department of Applied Mathematics, Faculty of Mathematics and Computer Sciences, Mathematikon, University Heidelberg, Im Neuenheimer Feld 205, D-69120 Heidelberg, Germany; friedmann@iwr.uni-heidelberg.de; 3 Institute of Hydrogeology, Engineering Geology and Applied Geophysics, Faculty of Science, Charles University, Albertov 6, 128 43 Praha 2, Czech Republic; martin.lanzendorfer@natur.cuni.cz; 4 Department of Pharmacology and Toxicology, Faculty of Pharmacy, Charles University, Heyrovského 1203, 500 05 Hradec Kralove, Czech Republic; pavek@faf.cuni.cz

**Keywords:** Pregnane X receptor, gene regulation, mathematical models, simulation

## Abstract

The pregnane X receptor (PXR) is a drug/xenobiotic-activated transcription factor of crucial importance for major cytochrome P450 xenobiotic-metabolizing enzymes (CYP) expression and regulation in the liver and the intestine. One of the major target genes regulated by PXR is the cytochrome P450 enzyme (CYP3A4), which is the most important human drug-metabolizing enzyme. In addition, PXR is supposed to be involved both in basal and/or inducible expression of many other CYPs, such as CYP2B6, CYP2C8, 2C9 and 2C19, CYP3A5, CYP3A7, and CYP2A6. Interestingly, the dynamics of PXR-mediated target genes regulation has not been systematically studied and we have only a few mechanistic mathematical and biologically based models describing gene expression dynamics after PXR activation in cellular models. Furthermore, few indirect mathematical PKPD models for prediction of CYP3A metabolic activity in vivo have been built based on compartmental models with respect to drug–drug interactions or hormonal crosstalk. Importantly, several negative feedback loops have been described in PXR regulation. Although current mathematical models propose these adaptive mechanisms, a comprehensive mathematical model based on sufficient experimental data is still missing. In the current review, we summarize and compare these models and address some issues that should be considered for the improvement of PXR-mediated gene regulation modelling as well as for our better understanding of the quantitative and spatial dynamics of CYPs expression.

## 1. Introduction

Nuclear receptors (NRs), which are ligand- or hormon-activated transcription factors, are essential in the organism in many processes, such as hormonal regulation, homeostasis of endogenous compounds and ions, the regulation of both intermediary and xenobiotic metabolism, and the regulation of cell differentiation, development, and proliferation [1]. Furthermore, these receptors are associated with numerous health conditions and unbalanced physiological processes, for instance cardiovascular diseases, inflammation, neurodegenerative or liver deseases, and cancer. Highly relevant diseases and pathological conditions, such as obesity, diabetes mellitus type II, atherosclerosis, and hyperlipidemia, can be the results of a dysfunction in any of the processes regulated by NRs [2,3,4,5].

NRs are proteins that share a common evolutionary history and similar sequence features at the protein level, primarily in their DNA-binding domain and to a lesser degree in ligand-binding domains [1,6,7,8].

The pregnane X receptor (PXR) (or NR subfamily 1, group I, member 2, NR1I2), the Constitutive androstane receptor (CAR) NR1I3, and the vitamin D receptor (VDR) NR1I1 represent group I of the subfamily 1 of NRs [6]. Several NRs have been characterized in the past as receptors for xenobiotics (so-called “xenosensors”) regulating the detoxification of xenobiotics or toxic endobiotic compounds, such as primary bile acids. Nowadays, Pregnane X receptor (PXR), Constitutive androstane receptor (CAR), and Aryl hydrocarbon receptor (AHR, a member of the family of basic helix-loop-helix transcription factors) are referred to as xenosensors critical to the regulation of key xenobiotic-metabolizing enzymes of both phase 1 and 2 of detoxification as well as of some transporters contributing to the elimination of toxic compounds from the body [9,10]. Interestingly, the last member of nuclear receptor subfamily 1 group I, the vitamin D receptor, being considered a classic vitamin or hormone receptor, also regulates numerous drug-metabolizing enzymes. Additionally, vice versa, PXR, which is considered only as a receptor of xenobiotics, can bind numerous endogenous compounds and significantly regulate glucose and lipid metabolism [11]. This documents that there is significant interplay of hormonal and detoxification metabolism regulation as well as parallel regulation of both intermediary and xenobiotic metabolism by PXR.

Until the discovery and characterization of the xenobiotic NR pregnane X receptor in 1998, it has been complicated to describe the molecular mechanisms by which drugs regulate enzyme and transporter expression [12,13,14].

The PXR is a critical NR that serves mainly in xenobiotic regulation. Nowadays, PXR is believed to be a part of the body’s adaptive defense mechanisms that play a vital role in the detoxification and clearance of toxic compounds and foreign chemicals. It acts mainly as a biological sensor of hydrophobic xenobiotics. It regulates, among other things, the expression of the CYP3A4 gene, which is involved in detoxification of almost 50% of all drugs [15]. Further studies proved the role of PXR in the regulation of many other important genes that influence different aspects of metabolism in the human body, such as CYP2B6, CYP2C9, CYP2C29, CYP3A5 and CYP3A7, UGT1A1, and ABCB1 (MDR1) [16].

The main difference between the pregnane X receptor and other NRs is the vast diversity of PXR’s ligands, including prescription drugs, herbal medicines, endobiotics, dietary supplements, and environmental pollutants [11,17,18].

An understanding of the regulation of drug-metabolizing enzymes expression and activity in response to drug administration, leading to drug–drug interactions (DDIs), is nowadays an essential issue in rational pharmacotherapy. These interactions influence the degree of absorption or elimination of medications and can modify the therapeutic or toxicological response to a drug [19,20]. These DDIs are risky mainly when coadministered drugs have narrow therapeutic windows or if they are intended to be therapy for life-threating diseases. Any decrease in plasma levels of these drugs due to DDI caused by an augmented metabolism via nuclear receptor activation can result in failure of therapy. Since PXR ligands can be found in almost all therapeutic groups of medication, we can hardly avoid DDI-based activation of PXR. Systematic quantitative description of CYP3A4 expression and regulation could significantly contribute to the rational and more efficient pharmacotherapy of many drugs, in particular with respect to dosing regimens.

However, no systematic algorithm or widely accepted predictive model to evaluate, quantify, and eliminate DDIs have been developed so far. Since many PXR ligands can also function as reversible or irreversible inhibitors of CYPs, the overall effects of PXR ligands should be considered with respect to DDIs, which challenges us to build a suitable quantitative model of DDIs [21].

In the current review, we summarize quantitative models that describe PXR-mediated regulation of cytochrome P450 3A gene expression with several experimental models for primary human or rat hepatocyte cell lines. In particular, we focus on mechanistically and biologically based cellular models that concentrate on molecular aspects of PXR-mediated regulation of the most important target gene, CYP3A4, or its rodent orthologues.

## 2. Pregnane X Receptor Characterization

The human PXR is the product of the NR1I2 gene, which is located on chromosome 3, locus 3q11–q13.3. The NR1I2 gene comprises 10 exons separated by nine intronic regions [22,23]. Several protein isoforms of the human PXR have been characterized with differential transcriptional activity. The transcript variants 1 (PXR1) and 2 (PXR2) respond to an agonist by activating target gene expression, while transcript variants 3 (PXR3) and the truncated variant 4 (PXR4) do not induce target gene expression [24]. The identification of PXR as a xenosensor presents a molecular basis for the species specificity of the induction of the CYP3A genes [25]. High homology (95% at the amino acid level) has been reported between human PXR (hPXR) and mouse PXR (mPXR) in the DNA-binding domain (DBD) [26]. Thus, they can share PXR binding sites found in promoters of both the human CYP3A (CYP3A4, CYP3A5, and CYP3A7) and rodent Cyp3a (e.g., Cyp3a11) genes. Nevertheless, the homology is much lower (73% at the amino acid level) in the ligand-binding domain (LBD), which may illustrate the ligand specificity between these receptors. Furthermore, the X-ray crystal structure analysis of the PXR LBD supports this notion [27]. There are significant variations between PXR species regarding PXR activators. The ligand specificity of hPXR is very broad, mainly for large ligands, such as rifampicin, whereas mPXR has a narrower ligand specificity. For instance, mPXR is more sensitive to pregnenolone 16α-carbonitrile than hPXR, while hyperforin and rifampicin can activate hPXR more than mPXR [28]. Additionally, it has also been reported that dexamethasone is a more potent ligand for mPXR than for the human receptor [29,30]. PXR has a spherical ligand-binding pocket, which has been shown to be at least twice as large as those of the other steroid hormone or retinoid receptors [27]. The PXR ligand-binding pocket was found to be hydrophobic and flexible. Therefore, these structural properties may have accounted for the high ability of this receptor to recognize a diverse range of xenobiotics [27]. The species origin of the PXR receptor, rather than the promoter structure of CYP3A genes, dictates the species-specific pattern of CYP3A inducibility [31]. These results have encouraged the creation of “humanized” hPXR transgenic mice, where the human counterpart hPXR genetically replaces the mouse PXR in the liver [26].

### 2.1. PXR Localization

PXR expression occurs mainly in the liver, intestine, and to a lesser degree in the kidney. Minor expression of hPXR/mPXR mRNAs may appear in other tissues, such as the lung, stomach, peripheral blood monocytes, uterus, ovary, breast, adrenal gland, bone marrow, and some regions of the brain [11,16].

**Figure 1:**
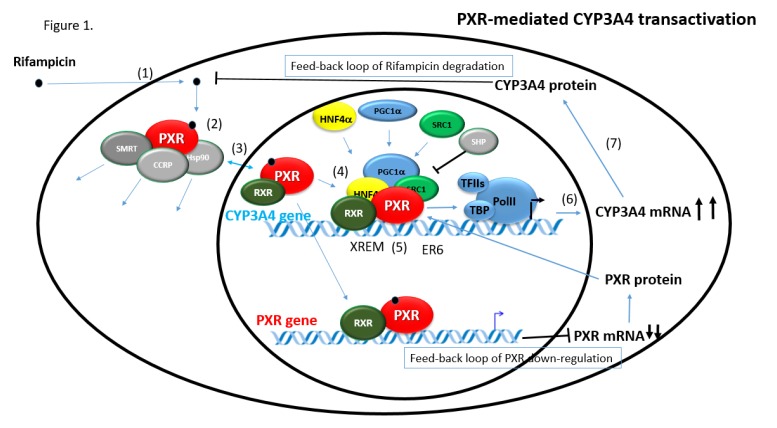
Schematic picture of pregnane X receptor (PXR)-mediated regulation of CYP3A4 gene expression. In the regulation, two feedback loops are involved. First, PXR triggers CYP3A4 mRNA transcription resulting in CYP3A4 protein synthesis in the cytosol. Subsequently, cytosolic CYP3A4 metabolically inactivates rifampicin, a substrate of CYP3A4. In the second feedback loop, activated PXR downregulates its own expression via the PXR (NR1I2) gene promoter. A potential third proposed feedback loop (not shown) supposes that PXR downregulates Small heterodimer partner (SHP) expression. SHP is a corepressor of PXR [49]. (**1**) passive diffusion of a ligand into cells; (**2**) binding to PXR that forms a cytoplasmic complex with chaperones (Hsp90), CCRP, and SMRT. The binding to a ligand promotes release of these proteins from PXR; (**3**) cytoplasm-nuclear translocation; (**4**) binding of coactivators and other nuclear receptors (such as RXRα) to form a transcriptional complex; (**5**) binding of the complex to PXR response elements in target genes’ promoter regions; (**6**) recruitment of transcription machinery factors and mRNA synthesis; (**7**) export of mRNA into cytosol and translation. HNF4α, hepatocyte nuclear factor 4α, a nuclear receptor that coactivates human PXR (hPXR); PGC1α, Peroxisome proliferator-activated receptor gamma coactivator 1-alpha, a coactivator of PXR; PolII, RNA polymerase II; TBP, TATA-binding protein; TFII, transcription factor II D.

The mPXR is primarily located in the cytosol of untreated liver cells, where mPXR binds with the cytoplasmic CAR retention protein (CCRP) and the heat shock protein 90 (Hsp90) to form a protein complex [11]. The latter retains the cytosolic localization of PXR. When the ligand binds to mPXR, the PXR dissociates from the multi-protein complex and translocates to the nucleus in mouse hepatocytes to regulate gene transcription [32,33] (Figure 1). Nevertheless, nuclear localization of hPXR has been found in mammalian tumor-derived cell lines [34].

### 2.2. PXR Transcriptional Machinery

As a typical NR, PXR has the DBD at the *N*-terminus and the LDB at the *C*-terminus. The DBD is capable of binding to regulatory DNA sequences [14]. Typically, there are two transcriptional activation domains in a nuclear receptor: the activation function 1 (AF-1), which resides in the *N*-terminal domain, and the AF-2, which is present in the *C*-terminal portion of the LBD. In the case of PXR, only one AF-2 is present in the LBD domain. The LDB has many functions, including ligand binding, dimerization, transcriptional activation, and interaction with transcriptional cofactors. AF-2 is the helix at the *C*-terminus that controls transcriptional activation through coactivators by conformational rearrangements of LBD or gene suppression through transcriptional corepressors recruitment [35,36].

The activation of nuclear receptors mostly takes place in the cytoplasm, where unliganded nuclear receptors typically reside in the complex with chaperones (such as Hsp90) and with co-chaperone CCRP. The schematic of PXR nuclear receptor activation is shown in Figure 1. Xenobiotics bind to the LBD of the PXR after they enter the cell. The binding of a ligand to the LBD results in a conformational change in the AF-2 that disrupts interactions with transcriptional corepressor proteins, such as NCoR and SMRT, triggers the release of the co-chaperone complex, and permits cytosolic-nuclear translocation of liganded PXR along cytoskeletal tracks toward the nucleus. Since the molecular weight of PXR is about 50 kDa, the nucleocytoplasmic transport is specifically regulated by an energy-dependent reaction (Nigg, 1997; Mattaj and Engelmeier, 1998). In the nucleus, formation of heterodimers with another NR called Retinoid X receptor-α (RXRα) and interactions with transcriptional coactivator proteins, such as members of the p160/steroid receptor coactivator (SRC) family, take place [8,33,37]. Afterwards, the PXR/RXRα heterodimer binds to the DNA through a pregnane X receptor response element (PXRE) and promotes the transcription of the target genes’ mRNAs [15,38]. These mRNAs then move to the cytoplasm, where they are subjected to translation into proteins which, as a result, do their function in metabolizing xenobiotics that invaded the cell [39].

In the review by Moore et al. [7], the authors reviewed some of the major coactivators and corepressors involved in the PXR regulation of drug-metabolizing enzymes and transporters. Steroid receptor coactivators 1 (SRC1/NCOA1) and 2 (SRC2/GRIP1), the NR interacting protein 1 (NRIP1/RIP140), the peroxisome proliferator-activated receptor gamma coactivator α (PGC-1α), and the Forkhead transcription factor FKHR (FOXO1) were identified as PXR coactivators [40]. Small heterodimer partner (SHP/NC0B2) and NR corepressor 2 (NCoR2/SMRT) were determined to be corepressors [40,41,42]. According to [41], in HepG2 cells, SHP hinders PXR binding to DNA in a ligand-dependent manner. Signaling cascades play a role in regulating PXR activity. In particular, the stimulation of PXR-mediated CYP3A induction by protein kinase A (PKA) has been shown [43] and PXR was reported to be phosphorylated by PKA in vitro. However, protein kinase C (PKC) was found to be a PXR signaling repressor [8,44]. The degradation of PXR via proteasomes is targeted by conjugation with a polyubiquitin chain. This “ubiquitination” process is governed by a sequential pathway of three different enzymes (E1, E2, and E3) and results in covalent binding of ubiquitin to PXR [45]. Other papers reported the involvement of acetylation in regulating PXR function [46,47]. Acetylation represents one of the major mechanisms for post-translational modification of proteins, including PXR [48]. PXR can also be regulated by other different signaling pathways, such as SUMOylation [8].

### 2.3. PXR Target Genes Regulation

PXR is currently regarded as a master transcription factor for xenobiotic- and drug-inducible expression of key genes that encode members of the phase I and phase II metabolic enzymes and drug transporters. Additionally, previous reports suggest that PXR plays an integral role in endobiotic metabolism by regulating important genes which have major roles in glucose, lipid, and bile acid metabolism [11]. A recent paper documented that PXR upregulates 164 genes, but downregulates the expression of 334 genes, in primary human hepatocytes [50].

#### 2.3.1. CYP3A4 Gene Regulation via PXR

*Trans*- as well as *cis*-regulator transcriptional elements involved in CYP3A4 gene expression have been comprehensively studied in the past. Apart from the basal promoter with an ER6 response element, the distal xenobiotic responsive enhancer module (XREM) is the key gene expression enhancer controlled by PXR. Depending on the species, these elements consist of DR3 motifs or ER6 motifs in the CYP3A4 gene promoter and function in a tissue-specific manner [15,51].

#### 2.3.2. PXR-Mediated Regulation of Other Target Genes

In addition to the CYP3A4 gene, PXR was characterized as an important metabolic regulator for the induction of the CYP2B6, CYP2C8/9, CYP2C19, CYP3A4/5/7, and CYP2A6 cytochrome P450 enzymes, phase II enzymes, such as UDP-glucuronyltransferases and sulfotransferases, and the upregulation of transporters, such as ABCB1/P-glycoprotein.

In the paper by Goodwin [52], it was shown that PXR is capable of activating the phenobarbital-responsive enhancer module region of the CYP2B6 gene. CYP2C8 and CYP2C9 are regulated by PXR via common NR AGGTCA-based DNA response element motifs, including everted repeats spaced by six base pairs (ER6) [53]. The P-glycoprotein transporter seems to be regulated by PXR through a DR4 motif in the upstream enhancer at about −8 kilo base pairs from transcription start [54]. In addition to the P-glycoptotein transporter, multidrug resistance protein 2 (MRP2, ABCC2), multidrug resistance-related protein-3 (MRP3, ABCC3), and organic anion transporter polypeptide-2 (human SLC22A7, mice oatp2) are regulated by PXR activation [54,55,56,57].

## 3. Ligands of the Pregnane X Receptor

Ligands that activate PXR include glucocorticoids, glucocorticoid receptor antagonists, antibiotics, antifungals, statins, calcium channel blockers (CCA), pesticides, and herbal extracts [58,59]. PXR is also activated by the naturally occurring steroids 5α-pregnane-3,20-dione, progesterone, 17α-hydroxyprogesterone, 17α-hydroxypregnenolone, and corticosterone [60]. Hyperforin is another high-affinity PXR agonist and is found in St. John’s wort. Patients using this herbal remedy while taking other medications have reported drug–drug interactions [61]. Table 1 contains the main representative drugs that are potent ligands of hPXR [18].

## 4. Brief Overview of Physiological Functions of PXR

PXR is today known to have numerous functions for various physiological purposes. Regulation of detoxification enzymes also contributes to the elimination of toxic endogenous compounds. Bile acids are a family of endogenous PXR ligands identified shortly after PXR had been cloned. Accumulation of bile acids in the liver can cause hepatocyte toxicity, and impaired elimination of bile acids from the body can be accompanied with pruritus during cholestatic diseases. PXR has been reported to function as a lithocholic acid sensor and has a crucial role in the detoxification of cholestatic bile acids [62,63]. Therefore, rifampicin, a prototype PXR ligand, is now used to alleviate pruritus by augmentation of bile acids metabolism and elimination [64]. Accumulation of bilirubin in the blood may cause neurotoxicity. PXR has been shown to stimulate the expression of a variety of essential factors in the bilirubin clearance pathway [65,66].

In addition, PXR plays an important endobiotic role in vitamin K homeostasis [67] and in vitamin D catabolism through the upregulation of CYP3A4 and CYP24 [68,69]. Importantly, vitamin E [70,71], beta-carotene [72], and some other bile acids [62] have been proposed as putative endogenous PXR ligands, which indicates that the physiological function of the compounds could be partly mediated by PXR activation. Further work shows that activation of PXR by rifampicin decreases the activity of NF-κB, an essential regulator of inflammation and the immune response [73,74]. PXR has also been considered in tumor progression [75].

A broad area of physiological functions have been proposed for PXR with respect to lipid, cholesterol, and glucose metabolism regulation. For detailed information, refer to the recent reviews [11,76,77].

## 5. Mathematical Models of PXR Activation and PXR-Induced Gene Expression

### 5.1. Compartmental Models

The main regulatory role of nuclear receptors, such as PXR, is up- and downregulation of gene expression resulting from augmented transactivation or *trans*-repression of its target genes. Gene expression is the outcome of the perturbation of a hierarchically organized and highly controlled network of interacting factors in signaling pathways and gene regulation networks in the cells, where ligand-activated nuclear receptors respond to an external stimulus. This process is distributed into several cellular compartments, including the nucleus and the cytosol. To understand these sophisticated networks, it is often beneficial to study the overall system by computational techniques based on complex biological knowledge, i.e., by a systems biology approach with a (semi-)mechanistic model rather than individual processes .

In a systems biology approach, the quantitative relationships in biologic systems are conveniently expressed by mathematical models. The important advantage of the models is that we can simulate different processes within different intervals, follow the dynamics of output parameters, and examine the effects of different parameters on different endpoints. Classical pharmacokinetic compartmental models, for example, describe the way one or several substances are distributed among distinct parts of a system through time. The system is conceptualized as consisting of one or more compartments, with the actual amount of each substance being represented in each compartment. The model then characterizes the transport of the substances from one compartment to another and/or the interaction of the substances inside a compartment, where any concentration inhomogeneity is ignored. Among the pharmacokinetic models, there are three main types: compartmental models, physiologically based models, and non-compartmental models [78]. Compartmental models represent the traditional and the most widely used approach in physiologically based pharmacokinetic (PBPK) and pharmacokinetic/pharmacodynamic (PK/PD) modelling [79].

The compartments are usually related to different sections of a body (such as organs) or different organelles of a cell type, each characterized by its volume of distribution (which may coincide with physiological volumes), within which the concentration of a drug is assumed to be homogeneous and to vary through time only. The mathematical description of the transport and interaction in time then consists of a system of ordinary differential equations (ODEs). Some of the parameters of the system of ODEs may be found in the literature and some donor-dependent parameters may be measured or derived directly. However, in practice, several parameters of the system of ODEs usually need to be estimated from in vitro or in vivo experiments through curve fitting. This can lead to several modelling stages: the parameters of a first simple model are fitted and the model is evaluated using obtained experimental data. The result may indicate where the model needs to be refined and an advanced model can be built with additional parameters, which in turn need to be fitted.

Importantly, the most useful technique for modelling the dynamics of gene network regulation over time employs compartmental modelling [80]. Among NRs, which regulate detoxification or metabolic enzymes, the glucocorticoid receptor (GR) seems to be the only receptor that has been extensively studied, and numerous mathematical models of several generations have been published to simulate its *trans*-activation in its target genes’ regulation [81]. Notably, by employing mathematical models, both ligand-dependent downregulation and circadian oscillation of GR receptor expression can be simulated and predicted [82]. Very recently, a multiscale pharmacodynamic model was developed to characterize the overall GR-mediated effects on transcriptome, proteome, and enzymatic activities after a single dose of methylprednisolone [83]. In the work, the authors also considered post-transcriptional processes in their mechanistic mathematical model that control some GR target gene expression.

In addition to mechanistic and biologically-based models, indirect response models and PK/PD modelling have been used to describe the effect of ligands of some nuclear receptors. Recently, population PK-PD models have been used to describe the exposure-response relationship between plasma rifampicin and 4β-hydroxycholesterol, an endogenous product of CYP3A [84,85]. Similarly, an indirect PK/PD response model has been built for the peroxisome proliferator-activated receptor gamma (PPARγ) agonist pioglitazone in vivo [86]. In the NONMEM model, a plasma glucose lowering effect was selected as a surrogate for an anti-diabetic effect of the drug. In another clinical study, a PK/PD model has been built to model the side effects of LY500307, a highly selective estrogen receptor β (ERbeta) agonist [87].

In the case of PXR, only three quantitative mechanistic mathematical models of PXR-mediated human CYP3A4 gene regulation in the liver have been published in the literature that incorporate data from humans or from human models [88,89,90]. In addition, one further model described rodent PXR regulation of its target genes (CYP3A1/2) in rats or in primary rat hepatocytes with the rodent PXR ligands dexamethasone, lithocholic acid, and pregnenalone-16α-carbonitrile [91]. Finally, Kolodkin et al. recently published an in silico model of the dual effects of the stress hormone cortisol on both PXR and glucocorticoid receptors activation [89]. The following table summarizes the application characteristics of the published models and specifies the software used for in silico computations (Table 2).

For the first model in the table, we display below the corresponding system of ordinary differential equations explicitly to give an impression of the arising type of equations. For the other models, the arising systems of ordinary differential equations are more complicated, with their explicit form sometimes given in the corresponding supplementary material or merely indicated with a diagram created by the used software. For the first model, the equations are:

(1) $$\frac{d\;X_{ext}\left(t\right)}{dt}=d\left(t\right)-k_{imp}X_{ext}\left(t\right)+k_{exp}X_{int}$$

(2) $$\frac{d\;X_{int}\left(t\right)}{dt}=k_{imp}X_{ext}\left(t\right)-k_{exp}X_{int}\left(t\right)-k_{assoc}X_{int}\left(t\right)\left(S_{PXR}-PR\left(t\right)\right)-k_{met}CYP3A4\left(t\right)X_{int}\left(t\right)+k_{dis}PR\left(t\right)$$

(3) $$\frac{d\;PR\left(t\right)}{dt}=k_{assoc}X_{int}\left(t\right)\left(S_{PXR}-PR\left(t\right)\right)-k_{dis}PR\left(t\right)$$

(4) $$\frac{d\;mRNA\left(t\right)}{dt}=k_{mRNA}PR\left(t\right)-k_{mRNA,deg}mRNA\left(t\right)+p_{mRNA,back}$$

(5) $$\frac{d\;CYP3A4\left(t\right)}{dt}=k_{cyp}mRNA\left(t\right)-k_{cyp,deg}CYP3A4\left(t\right).$$

Here, the variables $X_{ext}\left(t\right)$, $X_{int}\left(t\right)$, PR(t), mRNA(t), and CYP3A4(t) denote the concentrations of, respectively, the xenobiotic outside the cell, the xenobiotic inside the cell, the PXR/RXR heterodimer, mRNA, and CYP3A4; and d(t) is the time-dependent dosing function. The parameters $k_{imp}$, $k_{exp}$, $k_{dis}$, $k_{mRNA}$, $k_{mRNA,deg}$, $k_{cyp}$, and $k_{cyp,deg}$ are first order constants for, respectively, the import and export of the xenobiotic, the heterodimer dissociation, the transcription rate of mRNA, the degradation coefficient of mRNA, the translation rate of CYP3A4, and the degradation coefficient of CYP3A4. $k_{assoc}$ is the association rate constant for the formation of PXR/RXR heterodimer, $S_{PXR}$ is the total system PXR concentration (bound and free), which is assumed to be constant, $k_{met}$ is a second-order metabolic constant, and $p_{mRNA,back}$ is the background production rate for mRNA. A more detailed description of the processes represented by the five equations, including an explanatory modelling figure, is given in the next section.

As seen above, the model considers PXR expression, which is denoted as *S_PXR_*. Importantly, this parameter can significantly vary in a human population due to the PXR expression variability. Highly variable expression of PXR mRNA and protein has been published many times before [92,93] and over 100-fold variability in PXR mRNA expression in both males and female can be found in larger human populations (GTEx Portal, Broad Institute of MIT and Harvard University). Numerous single-nucleotide polymorphisms of the NR1I2 gene have been described to affect PXR expression, having clinically significant manifestations or being associated with disease progression [94,95] (PharmGKB Database). In addition, different transcription variants can be present in different humans having different transcriptional activities [24]. Thus, the incorporation of the mechanistic and molecular aspects into a model can help us to reflect interindividual PXR expression and activity.

### 5.2. Models of PXR-Mediated Regulation of CYP3A Enzymes

#### 5.2.1. Model by Luke et al.

**Figure 2:**
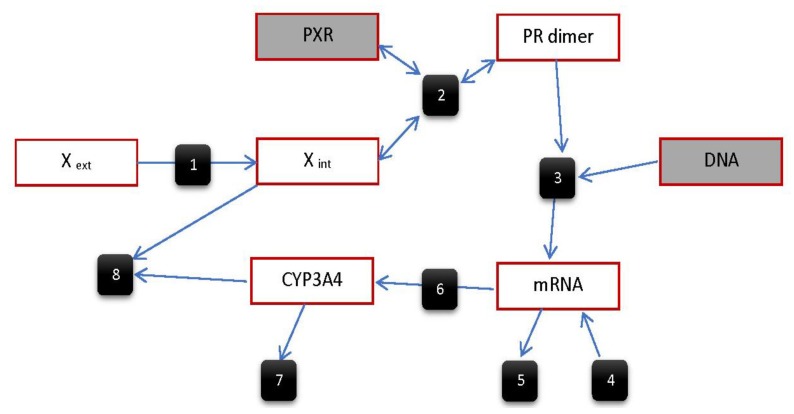
Schematic representation of the modelled PXR-mediated processes. Numbered circles represent the following reactions: (**1**) the Xenobiotic enters the cell; (**2**) PXR binds to the xenobiotic, leading to formation of PXR/RXRα heterodimer; (**3**) PXR/RXRα dimer binds to DNA, increasing transcription; (**4**) mRNA background production; (**5**) degradation of mRNA; (**6**) the translation of mRNA forms protein; (**7**) degradation of CYP3A4 protein; (**8**) the CYP3A4 protein metabolizes the xenobiotic [90].

The first quantitative (semi-)mechanistic biologically based compartmental model for PXR-mediated human CYP3A4 gene regulation has been described in the report by Luke et al. [90]. A schematic representation of the modelled processes is given in Figure 2, which can also be found in Luke et al. [90]. 

The model considers the standard processes connected with PXR signaling and PXR-mediated transactivation of its target genes, including the entry of rifampicin, a model PXR ligand, into hepatocytes by passive diffusion (described by Fick’s law), ligand binding to PXR leading to the formation of PXR/RXR heterodimer in the nucleus, binding of heterodimer to DNA, mRNA transcription, and translation of mRNA to CYP3A4 proteins (described as a first-order process).

In addition, the model incorporates mRNA background production and degradation, CYP3A4 degradation, and, importantly, a feedback CYP3A4-mediated degradation of rifampicin. First-order kinetics are used to model the diffusion between compartments, heterodimer dissociation, mRNA transcription and translation, degradation of mRNA and CYP3A4, and the formation of the heterodimer, while the degradation of rifampicin by CYP3A4 is modelled by second-order kinetics. Incorporation of zero-order kinetics of mRNA background production and first-order kinetics of CYP3A4 mRNA degradation enables us to model steady-state CYP3A4 mRNA levels. This is very convenient, since there is a constitutive CYP3A4 mRNA as well as CYP3A4 protein expression in normal hepatocytes. In addition, this allows us to use fold-induction data to describe upregulation (induction) of CYP3A4 mRNA or protein from in vitro cellular experiments. These data can be easily obtained using standard qRT-PCR or Western blotting methods. In contrast, absolute quantification of mRNA transcripts is always difficult and needs sophisticated instrumentation or calibration.

The corresponding system of ordinary differential equations for the model is depicted above.

Two forms of the model have been proposed in the work. The first one describes the situation in in vitro primary human hepatocytes in culture. It is composed of two compartments: the culture medium outside the plated cell culture (where rifampicin is present) and the interior of the plated cells. The model does not consider the compartmentalization of a cell into a nucleus and cytoplasm, which is a simplification that can lead to error outcomes since mRNA translocation from the nucleus into cytoplasm is an important process in gene regulation.

In the second model, the authors combined the cellular model with a model representing the human body in vivo. The model contains one compartment for blood (with a zero-order intravenous application of rifampicin and taking into account urinary excretion) and one for the liver. This model is parameterized with physiologic parameters, such as blood flow rate, liver and distribution volume, and the liver/blood partition coefficient.

The values necessary to run the models were taken from previously published papers. However, a small number of parameters (4 for the in vitro and 6 for the in vivo model) had to be estimated through least-squares curve fitting. In addition, a sensitivity analysis was performed and computed to assess the influence of the accuracies of the estimated parameters on the output concentrations. All computations were done using MATLAB^®^ (Math Works^®^, Natick, MA, USA) [97].

The models were validated through comparison with published data for rifampicin blood concentrations and CYP3A4 mRNA fold induction data measured in primary human hepatocytes. Whereas the model predictions for rifampicin concentrations in human plasma are acceptable, CYP3A4 mRNA expression profiles at early points modelled in the cellular model are discrepant in comparison with experimental data. Therefore, the authors modified the model and incorporated, through inserting an additional transit compartment, an artificial delay in CYP3A4 mRNA synthesis after PXR activation. In the modified setting, an improved fit of predicted CYP3A4 mRNA expression data with experimental data was observed. The authors suggest that the delay is caused by the fact that the model does not address cytoplasm-to-nucleus mRNA translocation as well as RNA processing, which could be carried out by defining an additional compartment for the nucleus. Moreover, when the dose-dependent induction of CYP3A4 mRNA was modelled, the predictions provided a fine fit with the collected data, but they did not approximate the data accurately at larger doses because a maximum of response was observed due to saturation.

This model thus introduced quantitative modelling of PXR-mediated regulation of the CYP3A4 target gene in a cellular model and enabled rough modelling with different parameters. In addition, the authors tried to scale the cellular model for a liver scenario employing hepatocellularity factor scaling. This attempt, however, was not further applied.

#### 5.2.2. Model by Yamashita et al.

In a paper by Yamashita et al. [88], another mechanism-based model for PXR-mediated human CYP3A4 gene regulation was developed. The paper contains in fact a collection of models for predicting DDIs caused by PXR activation.

First of all, the authors modelled the intracellular induction dynamics of CYP3A4 expression in primary human hepatocytes by three ODEs for the amount of activated PXR, the CYP3A4 mRNA level, and the CYP3A4 enzyme level. As in the previous model, it incorporates mRNA background production and degradation as well as CYP3A4 enzyme-mediated degradation of rifampicin as a negative feedback loop regulation. First-order kinetics are used to model PXR’s degradation, to model PXR-induced mRNA transcription and the degradation of mRNA, and to model the mRNA-activated production of CYP3A4 protein and its degradation. The action of rifampicin is not modelled based on diffusion from an outside compartment but using its affinity parameter (EC_50_ concentration) with respect to PXR, i.e., using a Hill equation. Zero-order kinetics are used for mRNA background production.

Employing the mathematical modelling and a large set of experimental data from the literature, the authors predicted degradation rate constants for CYP3A4 mRNA and proteins in primary human hepatocytes and showed a mechanistic application of the model in the examination of molecular aspects of PXR-mediated CYP3A4 regulation after a single-dose treatment.

Two extrapolations of this basic model are further presented: An indirect effect dynamic model, which assumes that hepatic CYP3A4 protein levels depend dynamically on the actual liver unbound fraction concentration of rifampicin (yielding just one ODE), and a static model, where hepatic CYP3A4 levels depend on the average steady state concentrations of rifampicin in plasma.

Moreover, a PBPK model for the clinical pharmacokinetics of rifampicin concentration in the blood and the liver is proposed in the paper. Employing the model, the authors simulate the fact that during repeated oral doses, rifampicin clearance is an accelerating nonlinear saturable process due to feedback PXR-mediated induction of rifampicin metabolism. This leads to mass-balance equations not only for rifampicin blood and liver concentrations, but for the inducible maximum elimination rate in the liver as well. The equation for rifampicin liver concentration contains, in addition to the terms in the work by Luke et al. [90], a Michaelis–Menten term and a repeated-dose term.

Computations in the report were performed in the multi-hierarchical physiology simulation platforms PhysioDesigner (for the PBPK models) and CellDesigner (for the intracellular simulation) [98]. They included the estimation of unknown model parameters. To cope with the influence of inter-donor variability of drug metabolism in hepatocytes, least-squares based analyses were carried out using the NONMEM software (Icon plc., Dublin, Ireland) [99].

This model demonstrates that rifampicin-induced drug–drug interactions can be extrapolated with acceptable accuracy using the developed models. Furthermore, the static model can be a tool to simulate DDI once the blood concentration of the inducer reaches steady-state after the administration of repeated oral doses.

#### 5.2.3. Model by Li et al.

A PK/PD model for rat CYP3A orthologues expression in the manuscript by Li et al. [96] describes PXR-mediated induction of CYP3A1/2 enzymes by the rat PXR ligand dexamethasone. It investigates the effect of PXR by monitoring in parallel both CYP3A1 and CYP3A2 mRNAs and proteins expression and by measuring the final total 6β-testosterone hydroxylation formation enzyme activity.

To model dexamethasone concentrations after intraperitonial administration (100 mg/kg) to rats, a two-compartment mammillary PBPK model with two ODEs for the blood and the liver was used. Similarly to the manuscript by Luke et al. (2010), it assumes zero-order drug absorption and comprises systemic (urinal) excretion, but it considers drug concentrations only. The link with PXR activity is indirectly modelled with a Hill equation giving, in dependency of liver dexamethasone levels, the relative concentration of DNA binding sites bound to dexamethasone–PXR complexes. As experimental data in rats suggest that CYP3A1 and CYP3A2 mRNA levels versus time are significantly delayed after dexamethasone application to rats, two parallel chains of transit compartments (filled with abstract substances called stimulation) were used. For CYP3A1, one transit compartment suffices to capture the delay, but CYP3A2 required eight transit compartments. The optimal number of transit compartments was found through stepwise addition or deletion of one transit compartment and was selected from the inflection point on an objective function value (presumably representing a measure of model quality, but this is not specified in the paper) versus compartment number curve.

Next, the stimulation concentration for the final transit compartment predicts mRNA levels using first-order kinetics. CYP3A1/2 mRNA are further assumed to be produced in the background by a zero-order rate constant and mRNA degradation to take place via a first-order process. CYP3A1/2 enzyme activity is then modelled with first-order constants for CYP3A1/2 degradation and synthesis via mRNA translation, where an amplification factor indicates that one copy of the mRNA can be translated into multiple copies of the protein. Finally, the computed protein concentrations of both CYP3A1 and CYP3A2 were combined to predict the resulting overall 6β-testosterone hydroxylation formation.

After fitting pharmacokinetic data, PK parameters were fixed and PD parameters were estimated using NONMEM software version 7.1.2. Applying these models to experimental data, the authors achieved a satisfactory fit of CYP3A1 and CYP3A2 mRNA profiles with parallel estimation of CYP3A1 mRNA and CYP3A2 mRNA delay. Similarly, both CYP3A1 and CYP3A2 protein dynamics with an appropriate lag time between mRNA synthesis and protein synthesis (about 12 h), as well as the CYP3A activity profile, were predicted with sufficient accuracy.

This model very well demonstrates the feasibility of in vivo PXR target gene induction modelling using PK/PD models and might be the most appropriate model that predicts the data rather accurately. This may be due to the fact that the authors used absolute concentration data (but not relative fold-upregulation data) for both CYP3A1/2 mRNA and CYP3A1/2 proteins in the work. The addition of a chain of transit compartments was essential to model the delay between mRNA transcription levels and drug plasma concentrations. Unfortunately, the report is based on animal data and parameters that cannot be easily extrapolated to humans.

#### 5.2.4. Model by Bailey et al.

Recently, Bailey et al. [91] developed a model to describe feedback and feedforward loops of the PXR-mediated transcriptional control of the rat CYP3A1 enzyme involved in steroid hormones deactivation. In the paper, the authors performed experiments with primary rat hepatocytes treated with different concentrations of rodent PXR ligands. They observed expression of whole-genome transcriptome using microarrays and expression of individual target genes using RT-PCR and Western blotting.

The authors formulated two models. The first one describes pregnenalone-16α-carbonitrile (PCN) as a xenobiotic PXR agonist, PXR-induced expression of its target gene CYP3A1, and downregulation of PXR transcript levels. The second model describes the effects of lithocholic acid as a common endogenous agonist for PXR, the farnesoid X-receptor, and the vitamin D receptor (VDR) on regulation of their target genes CYP3A1, Fibrinogen B, and CYP24, respectively. The effect on testosterone, cortisol, and progesterone metabolism was also included in the models. These in silico models were generated using CellDesigner and parameter values were taken from the literature if possible or estimated from experimental data.

The models show that biological response to chemicals occurs by the interaction of NRs via regulatory loops or through competition for ligands and response elements. The levels of PXR, controlled by the feedback loop, are reduced to approximately 50% of that seen with no agonist present. The model demonstrates that this feedback loop is an important factor to prevent hyperexpression of PXR target genes, such as CYP3A, which was confirmed in vitro and may help in explaining the robustness in steroid homeostasis within the cell. Thus, PXR might be regarded not only as a xenosensor but possibly also as an endosensor. The model helps us in understanding how the functions of PXR on xenobiotic and endobiotic metabolism are balanced to achieve both enough sensitivity to eliminate xenobiotics and at the same time maintain the hormonal homeostasis of the organism.

#### 5.2.5. Model by Kolodkin et al.

Kolodkin et al. [89] developed a model to describe the combined regulatory network and interaction of two NR’s in the human liver as a response to the stress hormone cortisol, a dual ligand of both PXR and glucocorticoid receptor (GR). As stress exposure causes transient blood cortisol spikes, the authors modelled the effect of cortisol on its two nuclear receptors. Cortisol is considered to be a high-affinity ligand for GR and a low-affinity ligand for PXR. Despite that the model represents only a small part of the whole biological system, excluding the possibility of changes in other proteins outside the model, it gives a realistic and complex view of the regulatory response to glucocorticoids in the human liver, including feedforward and feedback loop regulation.

The modelled GR-induced reactions are upregulation of PXR transcription [100], auto-downregulation of GR [101], and stimulation of the transcription of the classic marker gene tyrosine aminotransferase (TAT) [102], which represents the pharmacodynamic response to GR activation. As for the addressed PXR-induced signals, the formed PXR–ligand complex upregulates a second target gene set, including the marker gene CYP3A4 [103]. The reduction in levels of cortisol by CYP3A4 is modelled in the frame of a feedback loop. Reactions are described using various balance and rate equations. The resulting rather extensive and sophisticated signal regulatory network was constructed by using the CellDesigner software, with parameter values obtained or estimated based on the literature and de novo-generated data.

The model explains, among others, the important role of GR-induced upregulation of PXR. This upregulation allows PXR to bind to a multitude of low-affinity endogenous chemicals, all leading to increased PXR target genes, such as CYP3A4, and as a consequence stimulates the return to homeostasis. Thus, in spite of the low affinity of cortisol for PXR, PXR’s promiscuity, in combination with a GR-induced feedforward signal, makes it important for an appropriate response to stress challenge.

The model further describes an essential feature of the network: TAT levels, representing the pharmacodynamic response of GR, are significantly less sensitive to increased frequency of repeated stress episodes than the other modelled species. This demonstrates the ability of the network to adapt and thus limit the magnitude of the physiological response and the risk of disease progression. The model also predicts that there is a negligible difference between the stress response after repeated days with medium stress and the stress response following a weekend of no stress. In contrast, following a short vacation, the model predicts a muted response.

## 6. Discussion

Each of the described models has its own merits and restrictions. In this section, we will discuss some options to complement the existing models that address additional mechanistic questions or use different computational methodologies.

The currently published quantitative models of PXR-mediated regulation of its target genes use several approaches. Some models first describe PXR-mediated regulation in cellular models of hepatocytes and then aim to extrapolate the model to an in vivo situation based on indirect correlation of plasma PXR ligand concentrations with compartmental or whole body physiologically based pharmacokinetics models (PKPB) [88,89,90,91]. Some other models use in vivo data obtained in animals [96,104]. Some models are mechanistically based and consider the entry of a ligand into cells or cytoplasm-nuclear translocation of the liganded PXR [90], but others do not consider morphological factors of cells. Importantly, most of the models consider feedback (or feedforward) loops in PXR-mediated regulation of CYP3A genes, but only some of them can implement both of the critical feedback loops, i.e., CYP3A4-mediated degradation of a PXR ligand and PXR-mediated downregulation of PXR gene expression. Interestingly, some models use data obtained after the treatment of hepatocytes in one time interval with gradually increased concentrations of a PXR ligand [89,91].

All models studied the expression of a CYP3A orthologue as the most important target gene of PXR. Of note, Li et al. (2012) found that CYP3A1 and CYP3A2 genes and enzymes are differently regulated by PXR activation in rats, which clearly indicates that PXR target genes can have different time profiles of expression [96].

### 6.1. Feedback Loops

The papers of Kolodkin et al. and Bailey et al. included feedback and feedforward loops into the models. Importantly, PXR autoregulation resulting in PXR-mediated transrepression (downregulation), which was first proposed by Dr. Plant’s group [105], has been incorporated into their models.

This autoregulation mechanism has already been applied and demonstrated in the mathematical modelling of quantitative glucocorticoid receptor (GR)-mediated regulation. GR is another member of the nuclear receptor superfamily (NR3C1), which has very similar intracellular transcriptional machinery.

Glucocorticoids are an essential class of drugs indicated for the treatment of autoimmune diseases, such as rheumatoid arthritis. Recently, a fifth-generation model for the corticosteroid dynamic of GR activation has been reported. In the model, which is accompanied by comprehensive animal data, the authors incorporated cellular mechanistic events (including cytoplasm–nucleus recycling of GR) as well as feedback downregulation of GR by glucocorticoids [81]. According to the study, after a single bolus dose of glucocorticoids, the free cytosolic receptor levels fell immediately to zero and returned to baseline by 72 h. When two doses were given at 0 and 24 h, the second dose had a reduced effect due to the downregulation of the GR. Thus, less free cytosolic receptors were available for the next dose. This shows the importance of understanding the downregulation of NRs, including PXR, in multi-dose pharmacotherapy.

According to our knowledge, temporal data concerning the downregulation of PXR was not considered or used in any model. Baily et al. first proposed that PXR negative feedback downregulation of PXR expression attenuates the expression of the CYP3A gene as was shown using in silico modelling and a data set with only 48 h time-endpoint measurements.

**Figure 3:**
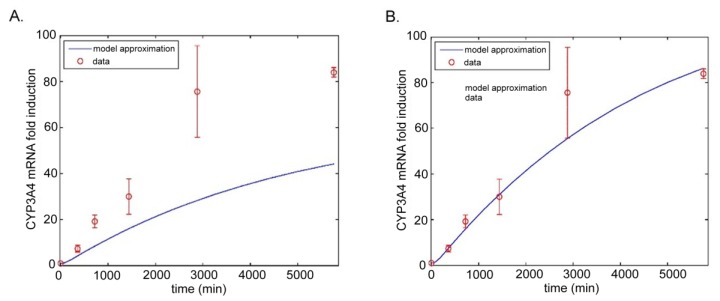
Time profiles of CYP3A4 mRNA induction in primary human hepatocytes modelled employing the model by Luke et al. 2010 with an estimated total (free and bound) intracellular PXR concentration of 10^−6^ μM (left, **A**.) and of 2 × 10^−6^ μM (right, **B**.). Rifampicin was applied at the concentration of 10 μM. The model was built in Matlab software version R2015.

We remark that PXR levels exhibit a large inter-donor variability caused by, among other things, differing PXR gene (NR1I2) genotypes, mutations, or variants. We demonstrate the importance of this issue in the validation of the model of Luke et al. with our own experimental data. In the experiment, primary human hepatocytes were treated with 10 µM of rifampicin. The expression level profiles of mRNA for the CYP3A4 enzyme were analyzed using the qRT-PCR method at 0, 6, 12, 24, 48, and 96 h from the beginning of the treatment. The measured average fold levels are displayed as circles in Figure 3 and the blue curves give the levels predicted by the model of Luke et al. [90]. In the left part of the figure (Figure 3A.), we used both the literature parameter values and the estimated parameter values reported by Luke et al. Clearly, our experimental data do not fit well with the predicted estimation. However, one of the estimated parameters is the total amount of PXR in the cell. Doubling the value of this estimated parameter resulted in satisfactory fitting as can be seen in the right part of the figure (Figure 3B.). This example clearly indicates that the mechanistic molecular-based model can be parametrized with individual parameters based on PXR gene (NR1I2) genotype or particular PXR expression.

In the paper by Yamashita et al. [88], the authors used PKPB modelling to analyze the auto-induction metabolic deactivation of rifampicin following repeated oral dosing of rifampicin (clinical data were published in the literature). Thus, the authors show that the feedback inhibition of the PXR ligand rifampicin by CYP3A4-mediated degradation is an important phenomenon that has to be considered in clinics.

**Figure 4:**
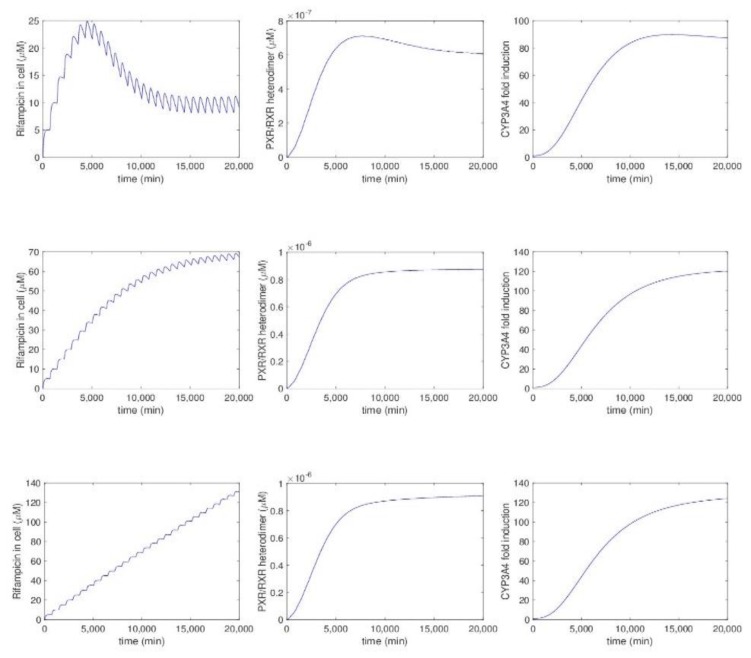
Time profiles of cellular rifampicin concentration, PXR/RXRα heterodimer abundance, and CYP3A4 protein induction in primary human hepatocytes with different rates of CYP3A4-mediated metabolism employing the model by Luke et al., 2010. Rifampicin was applied at the concentration of 10 μM every 12 h. In the upper figures, the feedback metabolism constant *kmet* equals *kmet* = 2.47 × 10^−4^, in the middle figures it equals *kmet* = 2.47 × 10^−5^, and in the lower figures it equals *kmet* = 2.47 × 10^−6^.

Unfortunately, there is little data on the simulation of PXR-mediated feedback loops after repeated dosage during longer time intervals (i.e., at least one week, as has been done for the GR in Kolodkin et al.). With our Matlab implementation of the in vitro model by Luke et al., we simulated fold-induction of the CYP3A4 enzyme with varying levels of PXR ligand metabolism by CYP3A4 for a period of about two weeks. We perturbed with one order of magnitude the estimated value of 2.47 × 10^−5^ in Luke et al. for *kmet*, a second-order metabolic constant indicating the feedback metabolism of rifampicin in cells. The amount of feedback metabolism seems to strongly influence rifampicin levels only (Figure 4). Predicted CYP3A4 fold-induction levels, especially for small values of *kmet*, seem very high and will probably not occur in in vivo situations; they might be caused by inaccurate estimation of the precise *kmet* value or of other parameters of the model. In a similar way, the influence could be predicted of the diffusion speed of the ligand into hepatocytes, of other aspects, or of interactions between them.

### 6.2. Extended Computational Methodologies

A major restriction in mathematical PK/PD modelling is the limited availability of reliable parameters for the model and complications with obtaining data. Some of these complications are connected with technical or methodological difficulties of data measurement in cellular models, in experimental animals, or in humans.

For the estimation of unknown parameters, the described models use curve fitting based on a comparison of observed with simulated concentration time profiles. Mathematically, the fitting consists of the minimization of a sum of squares cost function. Improved accuracy of the estimated parameters can be obtained with a Bayesian approach using Monte Carlo-type simulations. The basic idea is to generate a large number of random values for the model parameters with the help of a suitable pseudo-random number generator. Evaluation of the behavior of the model for random values can provide, using tools from statistics, relatively reliable information on general behavior (assuming that a large number of random values was generated). With the high performance of modern computer systems, such a process often has many advantages over laboratory experiments; it can also complement them or help us in the proper dosing of experiments based on pilot mathematical predictions. An example of a model employing such techniques for the simulation of the dynamic range of 4βHC as a biomarker for CYP3A4 induction and inhibition is presented in the report by Leil et al. [85] and in the paper by Jiang et al. [84].

The models we described are almost completely based on one of the following types of equations: First/zero order kinetics, mass-balance equations, the Hill equation, or Michaelis–Menten kinetics. These equations are considered appropriate for the involved reactions and appear to be used in PK/PD models for most other pharmacological applications as well. Nevertheless, the following remarks might be important. First, the justification for using a particular type of equation is sometimes missing. The process of ligand-binding, for instance, can be modelled with mass-action kinetics if this is known to be the only type of reaction taking place or with a Michaelis–Menten reaction, which is different. The Hill equation can be used to describe the cooperativity of binding to a receptor when more information is known (negative, positive cooperativity). These different modelling options lead to rather different mathematical equations. Different equations for the same portion of the signaling pathway may be due to the fact that some models simply use, from the outset, weaker assumptions than others. However, in some cases, the absence of a biological justification for the used modelling equations might suggest that they have been determined based on a trial-and-error basis; i.e., the equations leading to the best model for the given data were used. Although this seems to be the natural thing to do when no alternatives are available, it would be interesting to verify whether the presented models remain as accurate with different experimental data values for the same phenomena. In other words, the question is whether, in addition to the fitting of unknown parameter values to observed data, one actually fits the modeling equations to the observed data as well. Of course, one can find specific parameters for several models which represent the same given data. However, the goal is to understand and model the phenomena behind and make long-term predictions with the correct model. Here, a wrong model could give a different outcome.

Another example of ‘model fitting’, with respect to the number of compartments, is the definition of transit compartments to model a transport delay. The number of transit compartments is sometimes determined based on curve fitting to experimental data (e.g., in Li et al. [96]). In fact, introducing transit departments can be very convenient to precisely model reactions that are not understood in detail, i.e., when it is only observed that somewhere, something takes place that delays substance transport without knowledge of the type of reaction or of its location. In the latter case, the transit compartment does not have a precise physical place in the modelled process, which may sound a little counterintuitive. In other situations, complicated delaying processes are willingly replaced by a transit department to keep the overall model simple. If measurements are available, some phenomena in between can be neglected, and a model with limited equations can be better analyzed mathematically. For instance, the nuclear processes of the considered signaling pathway can be neglected because the concentrations leaving the nucleus can be measured. In the model of Luke et al. [90], the encountered delay is explained by the fact that the model ignores nuclear–cytoplasmic compartmentalization to incorporate mRNA nucleus-to-cytoplasm translocation. Hargrove [106] reports that indeed there is a delay before the appearance of mRNA in the cytoplasm that is due to the transcription of the gene, processing of the primary transcript, and nucleocytoplasmic transport [90].

Often, the unexplained or unmodelled reactions in transit compartments correspond, in reality, to the accumulation of a substance at some particular location before it is further transported. Protein synthesis occurs in ribosomes, which are located free in the cytosol or bound to the endoplasmic reticulum where synthesized proteins are localized [107]. Thus, CYP3A4 protein and its mRNA can accumulate near the reticulum. To gain more insight into *where* reactions take place and what their *local* effect is, an alternative to defining additional compartments is to incorporate spatial distribution in the substance concentration functions: The functions would be dependent not only on time but space-points as well. This requires an altogether different computational methodology, because differential equations for functions with more than one variable (so-called partial differential equations (PDEs)) have to be considered. Even if substances appear to often be homogeneously distributed, it can be beneficial to provide spatial resolution in compartments where experimental observations suggest heterogeneous distributions. For example, the model of Luke et al. [90] could be modified and the cytoplasm compartment modelled with PDEs (i.e., with spatial resolution) while the nucleus compartment is modelled as usual with ODEs. Mathematically, this leads to a mixed system of PDEs coupled with ODEs. The variables $X_{int}\left(t\right)$, PR(t), mRNA(t), and CYP3A4(t) in the equations displayed at the end of Section 5.1 will be dependent not only on a time variable *t*, but on a space variable *x* (with possibly three components in three dimensions) as well. For instance, the last equation then takes the form:

(6) $$\partial_{t}\;CYP3A4\left(t,x\right)=D_{CYP3A4}\Delta CYP3A4\left(t,x\right)+k_{cyp}mRNA_{cyp}\left(t,x\right)-k_{cyp,deg}CYP3A4\left(t,x\right),$$

where $D_{CYP3A4}$ is the diffusion coefficient (or matrix with anisotropic diffusion) for CYP3A4, Δ denotes the gradient, and $mRNA_{cyp}$ is the mRNA concentration in the cytoplasm.

PK/PD models including spatial resolution appear to have been proposed only rarely in other applications; some examples are reports by Claus et al. [108] and Friedmann et al. [109]. In the report by Thurley et al. [110], it is shown that high concentration gradients evolve despite fast diffusion. Here, the cytokine concentration is immediately distributed homogeneously in space, but the local reactions control the dynamics and lead to an inhomogeneous steady-state.

Because elevated drug concentrations are often toxic, it can be crucial to monitor not only the average drug level all over a compartment, but to detect possible localized maxima as well. Similarly, the exceeding of no-observed-adverse-effect levels in the context of drug–drug interactions should be detectable locally, inside compartments. The degree of heterogeneity of substance concentrations in an intracellular compartment can depend strongly on the speed of the diffusion, which is often inversely correlated with molecule sizes (e.g., larger molecules, such as rifampicin, penetrate the cell membrane slowly and incompletely). Cell shapes may have an influence as well. In the papers by Claus et al. and Friedman et al. [108,109], the authors found, in a different context, larger cytoplasmic concentration gradients in fibroblast cells than in regularly shaped ones. This can provide a delay or change in the nuclear processes. For these cases, the effect of the cell geometry on the nuclear processes is in fact higher than the effect of diffusion and elaborate diffusion measurements can be relaxed. For modelling signaling transduction in cells with spatial extensions, the more complex PDE modelling should be used. In general, the more complicated the signaling pathway dynamics is, in particular when feedback or feedforward loops are involved, the less obvious it is that homogeneity of concentrations can be assumed: Can a rather small heterogeneity in one of the substances cause a more significant variation in spatial distribution of other substances of interest? Can such a heterogeneity, in conjunction with intricate kinetics, bring about a delay? Even if spatial resolution may not seem necessary at first sight, it might reveal unexpected explanations for observed pharmacological phenomena.

## 7. Conclusions

Each of the described models has its own merits and restrictions. Currently, we have several more or less sophisticated models of PXR activation describing the cellular or in vivo scenario of target genes regulation. However, we need additional models based on robust experimental data that would comprehensively describe the molecular, organ, and whole-body aspects of PXR-mediated cytochrome P450 gene expression. In addition, all reports on modelling PXR focused only on the CYP3A4 gene and omitted other important CYP enzymes regulated by PXR. We do not have models that consider additional PXR ligands. Finally, PXR-mediated gene regulation has been exclusively modelled in the liver and the intestine has not been considered.

## Figures and Tables

**Table 1:** Representative drugs that are a potent ligand of human PXR.

Ligands with High Potency	Ligands with Medium Potency	Ligands with Low Potency
Rifampicin	Carbamazepine	Testosterone
Mifepristone	Dexamethasone	Indomethacin
SR12813	Zolpidem	Warfarin
Hyperforin	Rosiglitazone	Ethinyl estradiol
Nifedipine	Atorvastatin	Phenobarbital
Diltiazem	Omeprazole	Rosuvastatin
Clotrimazole	Loratadine	Tamoxifen
Rifabutine	Haloperidole	Saquinavir

**Table 2:** Properties of the described models.

Authors	PXR Ligand	Nuclear Receptor	PXR Target Gene Studied	Tested Model	Software Used	Data Sets
[90]	Rifampicin	PXR	CYP3A4	- Primary human hepatocytes - Two compartment model of with human central compartment and the liver (livers scaled from hepatocytes in culture using a scaling factor)	MATLAB	- CYP3A4 mRNA data in primary human hepatocytes from literature - human plasma concentration of rifampicin from literature
[88]	Rifampicin	PXR	CYP3A4	- Primary human hepatocytes - two compartment PBPK model	CellDesigner, PhysioDesigner, NONMEM	- CYP3A4 mRNA data in primary human hepatocytes from literature - human plasma concentration of rifampicin
[96]	Dexamethasone (a rat PXR ligand)	PXR	CYP3A1/2 (a rat CYP3A4 orthologue)	Rats - PK/PD model after i.p. application	NONMEM	Animal experiments, pharmacokinetic study with Sprague-Dawley rats receiving 100 mg/kg of dexamenthasone i.p.
[91]	PCN, LCA (rat PXR ligands)	PXR (VDR, FXR)	CYP3A1, CYP24, Fibrinogen B	Rats and human cell line Huh7	CellDesigner, MVSP	primary rat hepatocytes and Huh7 cells
[89]	Cortisol	PXR, GR	CYP3A4, TAT	Humans	CellDesigner	- primary human hepatocytes - data from literature

PK, pharmacokinetic; PD, pharmacodynamics; PBPK, physiologically based pharmacokinetic.

## Abbreviations

AF-1, AF-2	Activation function 1, 2
CAR	Constitutive androstane receptor
CCRP	Cytoplasmic CAR retention protein
CYP	Cytochrome P450
CYP3A4	Cytochrome P450 3A4
DBD	DNA-binding domain
DDI	Drug-drug interaction
GR	Glucocorticoid receptor
Hsp90	Heat shock protein 90
LBD	Ligand binding domain
NR	Nuclear receptor
ODE	Ordinary differential equation
PDE	Partial differential equation
PK/PD	Pharmacokinetic/pharmacodynamic
PAK	Protein kinase A
PXR	Pregnane X receptor
RXRα	Retinoid X receptor-α
SRC	Steroid receptor coactivator
TAT	Tyrosine aminotransferase
VDR	Vitamin D receptor
XREM	Xenobiotic responsive enhancer module

## References

[1] Germain P., Staels B., Dacquet C., Spedding M., Laudet V. (2006). Overview of nomenclature of nuclear receptors. Pharmacol. Rev..

[2] Halilbasic E., Baghdasaryan A., Trauner M. (2013). Nuclear receptors as drug targets in cholestatic liver diseases. Clin. Liver Dis..

[3] Imai Y., Youn M.Y., Inoue K., Takada I., Kouzmenko A., Kato S. (2013). Nuclear receptors in bone physiology and diseases. Physiol. Rev..

[4] Saijo K., Crotti A., Glass C.K. (2010). Nuclear receptors, inflammation, and neurodegenerative diseases. Adv. Immunol..

[5] Lazar M.A. (1999). Nuclear hormone receptors: From molecules to diseases. J. Investig. Med..

[6] Di Masi A., De Marinis E., Ascenzi P., Marino M. (2009). Nuclear receptors CAR and PXR: Molecular, functional, and biomedical aspects. Mol. Asp. Med..

[7] Moore D.D., Kato S., Xie W., Mangelsdorf D.J., Schmidt D.R., Xiao R., Kliewer S.A. (2006). International Union of Pharmacology. LXII. The NR1H and NR1I receptors: Constitutive androstane receptor, pregnene X receptor, farnesoid X receptor alpha, farnesoid X receptor beta, liver X receptor alpha, liver X receptor beta, and vitamin D receptor. Pharmacol. Rev..

[8] Smutny T., Mani S., Pavek P. (2013). Post-translational and post-transcriptional modifications of pregnane X receptor (PXR) in regulation of the cytochrome P450 superfamily. Curr. Drug Metab..

[9] Mackowiak B., Wang H. (2016). Mechanisms of xenobiotic receptor activation: Direct vs. indirect. Biochim. Biophys. Acta.

[10] Marino M., di Masi A., Trezza V., Pallottini V., Polticelli F., Ascenzi P. (2011). Xenosensors CAR and PXR at work: Impact on statin metabolism. Curr. Drug Metab..

[11] Pavek P. (2016). Pregnane X Receptor (PXR)-Mediated Gene Repression and Cross-Talk of PXR with Other Nuclear Receptors via Coactivator Interactions. Front. Pharmacol..

[12] Blumberg B., Kang H., Bolado J., Chen H., Craig A.G., Moreno T.A., Umesono K., Perlmann T., De Robertis E.M., Evans R.M. (1998). BXR, an embryonic orphan nuclear receptor activated by a novel class of endogenous benzoate metabolites. Genes Dev..

[13] Blumberg B., Sabbagh W., Juguilon H., Bolado J., van Meter C.M., Ong E.S., Evans R.M. (1998). SXR, a novel steroid and xenobiotic-sensing nuclear receptor. Genes Dev..

[14] Kliewer S.A., Moore J.T., Wade L., Staudinger J.L., Watson M.A., Jones S.A., McKee D.D., Oliver B.B., Willson T.M., Zetterstrom R.H. (1998). An orphan nuclear receptor activated by pregnanes defines a novel steroid signaling pathway. Cell.

[15] Goodwin B., Hodgson E., Liddle C. (1999). The orphan human pregnane X receptor mediates the transcriptional activation of CYP3A4 by rifampicin through a distal enhancer module. Mol. Pharmacol..

[16] Pavek P., Dvorak Z. (2008). Xenobiotic-induced transcriptional regulation of xenobiotic metabolizing enzymes of the cytochrome P450 superfamily in human extrahepatic tissues. Curr. Drug Metab..

[17] Kliewer S.A. (2003). The nuclear pregnane X receptor regulates xenobiotic detoxification. J. Nutr..

[18] Sinz M., Kim S., Zhu Z., Chen T., Anthony M., Dickinson K., Rodrigues A.D. (2006). Evaluation of 170 xenobiotics as transactivators of human pregnane X receptor (hPXR) and correlation to known CYP3A4 drug interactions. Curr. Drug Metab..

[19] Hedrich W.D., Hassan H.E., Wang H. (2016). Insights into CYP2B6-mediated drug-drug interactions. Acta Pharm. Sin. B.

[20] Prakash C., Zuniga B., Song C.S., Jiang S., Cropper J., Park S., Chatterjee B. (2015). Nuclear Receptors in Drug Metabolism, Drug Response and Drug Interactions. Nucl. Recept. Res..

[21] Wei Y., Tang C., Sant V., Li S., Poloyac S.M., Xie W. (2016). A Molecular Aspect in the Regulation of Drug Metabolism: Does PXR-Induced Enzyme Expression Always Lead to Functional Changes in Drug Metabolism?. Curr. Pharmacol. Rep..

[22] Hustert E., Zibat A., Presecan-Siedel E., Eiselt R., Mueller R., Fuss C., Brehm I., Brinkmann U., Eichelbaum M., Wojnowski L. (2001). Natural protein variants of pregnane X receptor with altered transactivation activity toward CYP3A4. Drug Metab. Dispos. Biol. Fate Chem..

[23] Zhang J., Kuehl P., Green E.D., Touchman J.W., Watkins P.B., Daly A., Hall S.D., Maurel P., Relling M., Brimer C. (2001). The human pregnane X receptor: Genomic structure and identification and functional characterization of natural allelic variants. Pharmacogenetics.

[24] Brewer C.T., Chen T. (2016). PXR variants: The impact on drug metabolism and therapeutic responses. Acta Pharm. Sin. B.

[25] Barwick J.L., Quattrochi L.C., Mills A.S., Potenza C., Tukey R.H., Guzelian P.S. (1996). Trans-species gene transfer for analysis of glucocorticoid-inducible transcriptional activation of transiently expressed human CYP3A4 and rabbit CYP3A6 in primary cultures of adult rat and rabbit hepatocytes. Mol. Pharmacol..

[26] Yan J., Xie W. (2016). A brief history of the discovery of PXR and CAR as xenobiotic receptors. Acta Pharm. Sin. B.

[27] Watkins R.E., Wisely G.B., Moore L.B., Collins J.L., Lambert M.H., Williams S.P., Willson T.M., Kliewer S.A., Redinbo M.R. (2001). The human nuclear xenobiotic receptor PXR: Structural determinants of directed promiscuity. Science.

[28] Iyer M., Reschly E.J., Krasowski M.D. (2006). Functional evolution of the pregnane X receptor. Expert Opin. Drug Metab. Toxicol..

[29] Scheer N., Ross J., Kapelyukh Y., Rode A., Wolf C.R. (2010). In vivo responses of the human and murine pregnane X receptor to dexamethasone in mice. Drug Metab. Dispos. Biol. Fate Chem..

[30] Moore L.B., Parks D.J., Jones S.A., Bledsoe R.K., Consler T.G., Stimmel J.B., Goodwin B., Liddle C., Blanchard S.G., Willson T.M. (2000). Orphan nuclear receptors constitutive androstane receptor and pregnane X receptor share xenobiotic and steroid ligands. J. Biol. Chem..

[31] Xie W., Barwick J.L., Downes M., Blumberg B., Simon C.M., Nelson M.C., Neuschwander-Tetri B.A., Brunt E.M., Guzelian P.S., Evans R.M. (2000). Humanized xenobiotic response in mice expressing nuclear receptor SXR. Nature.

[32] Kawana K., Ikuta T., Kobayashi Y., Gotoh O., Takeda K., Kawajiri K. (2003). Molecular mechanism of nuclear translocation of an orphan nuclear receptor, SXR. Mol. Pharmacol..

[33] Squires E.J., Sueyoshi T., Negishi M. (2004). Cytoplasmic localization of pregnane X receptor and ligand-dependent nuclear translocation in mouse liver. J. Biol. Chem..

[34] Saradhi M., Sengupta A., Mukhopadhyay G., Tyagi R.K. (2005). Pregnane and Xenobiotic Receptor (PXR/SXR) resides predominantly in the nuclear compartment of the interphase cell and associates with the condensed chromosomes during mitosis. Biochim. Biophys. Acta.

[35] Ding X., Lichti K., Staudinger J.L. (2006). The mycoestrogen zearalenone induces CYP3A through activation of the pregnane X receptor. Toxicol. Sci..

[36] Synold T.W., Dussault I., Forman B.M. (2001). The orphan nuclear receptor SXR coordinately regulates drug metabolism and efflux. Nat. Med..

[37] Johnson D.R., Li C.W., Chen L.Y., Ghosh J.C., Chen J.D. (2006). Regulation and binding of pregnane X receptor by nuclear receptor corepressor silencing mediator of retinoid and thyroid hormone receptors (SMRT). Mol. Pharmacol..

[38] Handschin C., Meyer U.A. (2003). Induction of drug metabolism: The role of nuclear receptors. Pharmacol. Rev..

[39] Kliewer S.A., Goodwin B., Willson T.M. (2002). The nuclear pregnane X receptor: A key regulator of xenobiotic metabolism. Endocr. Rev..

[40] Hariparsad N., Chu X., Yabut J., Labhart P., Hartley D.P., Dai X., Evers R. (2009). Identification of pregnane-X receptor target genes and coactivator and corepressor binding to promoter elements in human hepatocytes. Nucleic Acids Res..

[41] Ourlin J.C., Lasserre F., Pineau T., Fabre J.M., Sa-Cunha A., Maurel P., Vilarem M.J., Pascussi J.M. (2003). The small heterodimer partner interacts with the pregnane X receptor and represses its transcriptional activity. Mol. Endocrinol..

[42] Pavek P., Stejskalova L., Krausova L., Bitman M., Vrzal R., Dvorak Z. (2012). Rifampicin Does not Significantly Affect the Expression of Small Heterodimer Partner in Primary Human Hepatocytes. Front. Pharmacol..

[43] Ding X., Staudinger J.L. (2005). Induction of drug metabolism by forskolin: The role of the pregnane X receptor and the protein kinase a signal transduction pathway. J. Pharmacol. Exp. Ther..

[44] Ding X., Staudinger J.L. (2005). Repression of PXR-mediated induction of hepatic CYP3A gene expression by protein kinase C. Biochem. Pharmacol..

[45] Portbury A.L., Ronnebaum S.M., Zungu M., Patterson C., Willis M.S. (2012). Back to your heart: Ubiquitin proteasome system-regulated signal transduction. J. Mol. Cell. Cardiol..

[46] Staudinger J.L., Xu C., Biswas A., Mani S. (2011). Post-translational modification of pregnane X receptor. Pharmacol. Res..

[47] Biswas A., Pasquel D., Tyagi R.K., Mani S. (2011). Acetylation of pregnane X receptor protein determines selective function independent of ligand activation. Biochem. Biophys. Res. Commun..

[48] Wang C., Tian L., Popov V.M., Pestell R.G. (2011). Acetylation and nuclear receptor action. J. Steroid Biochem. Mol. Biol..

[49] Li T., Chiang J.Y. (2006). Rifampicin induction of CYP3A4 requires pregnane X receptor cross talk with hepatocyte nuclear factor 4alpha and coactivators, and suppression of small heterodimer partner gene expression. Drug Metab. Dispos. Biol. Fate Chem..

[50] Kandel B.A., Thomas M., Winter S., Damm G., Seehofer D., Burk O., Schwab M., Zanger U.M. (2016). Genomewide comparison of the inducible transcriptomes of nuclear receptors CAR, PXR and PPARalpha in primary human hepatocytes. Biochim. Biophys. Acta.

[51] Pavek P., Pospechova K., Svecova L., Syrova Z., Stejskalova L., Blazkova J., Dvorak Z., Blahos J. (2010). Intestinal cell-specific vitamin D receptor (VDR)-mediated transcriptional regulation of CYP3A4 gene. Biochem. Pharmacol..

[52] Goodwin B., Moore L.B., Stoltz C.M., McKee D.D., Kliewer S.A. (2001). Regulation of the human CYP2B6 gene by the nuclear pregnane X receptor. Mol. Pharmacol..

[53] Gerbal-Chaloin S., Pascussi J.M., Pichard-Garcia L., Daujat M., Waechter F., Fabre J.M., Carrere N., Maurel P. (2001). Induction of CYP2C genes in human hepatocytes in primary culture. Drug Metab. Dispos. Biol. Fate Chem..

[54] Geick A., Eichelbaum M., Burk O. (2001). Nuclear receptor response elements mediate induction of intestinal MDR1 by rifampin. J. Biol. Chem..

[55] Light R.W., Lee Y.C.G. (2016). Textbook of Pleural Diseases.

[56] Staudinger J., Liu Y., Madan A., Habeebu S., Klaassen C.D. (2001). Coordinate regulation of xenobiotic and bile acid homeostasis by pregnane X receptor. Drug Metab. Dispos. Biol. Fate Chem..

[57] Teng S., Jekerle V., Piquette-Miller M. (2003). Induction of ABCC3 (MRP3) by pregnane X receptor activators. Drug Metab. Dispos. Biol. Fate Chem..

[58] Lemaire G., Mnif W., Pascussi J.M., Pillon A., Rabenoelina F., Fenet H., Gomez E., Casellas C., Nicolas J.C., Cavailles V. (2006). Identification of new human pregnane X receptor ligands among pesticides using a stable reporter cell system. Toxicol. Sci..

[59] Willson T.M., Kliewer S.A. (2002). PXR, CAR and drug metabolism. Nat. Rev. Drug Discov..

[60] Goodwin B., Redinbo M.R., Kliewer S.A. (2002). Regulation of cyp3a gene transcription by the pregnane x receptor. Annu. Rev. Pharmacol. Toxicol..

[61] Watkins R.E., Maglich J.M., Moore L.B., Wisely G.B., Noble S.M., Davis-Searles P.R., Lambert M.H., Kliewer S.A., Redinbo M.R. (2003). 2.1 A crystal structure of human PXR in complex with the St. John’s wort compound hyperforin. Biochemistry.

[62] Staudinger J.L., Goodwin B., Jones S.A., Hawkins-Brown D., MacKenzie K.I., LaTour A., Liu Y., Klaassen C.D., Brown K.K., Reinhard J. (2001). The nuclear receptor PXR is a lithocholic acid sensor that protects against liver toxicity. Proc. Natl. Acad. Sci. USA.

[63] Xie W., Radominska-Pandya A., Shi Y., Simon C.M., Nelson M.C., Ong E.S., Waxman D.J., Evans R.M. (2001). An essential role for nuclear receptors SXR/PXR in detoxification of cholestatic bile acids. Proc. Natl. Acad. Sci. USA.

[64] Webb G.J., Rahman S.R., Levy C., Hirschfield G.M. (2018). Low risk of hepatotoxicity from rifampicin when used for cholestatic pruritus: A cross-disease cohort study. Aliment. Pharmacol. Ther..

[65] Klaassen C.D., Slitt A.L. (2005). Regulation of hepatic transporters by xenobiotic receptors. Curr. Drug Metab..

[66] Wang J., Dai S., Guo Y., Xie W., Zhai Y. (2014). Biology of PXR: Role in drug-hormone interactions. EXCLI J..

[67] Tabb M.M., Sun A., Zhou C., Grun F., Errandi J., Romero K., Pham H., Inoue S., Mallick S., Lin M. (2003). Vitamin K2 regulation of bone homeostasis is mediated by the steroid and xenobiotic receptor SXR. J. Biol. Chem..

[68] Zhang B., Xie W., Krasowski M.D. (2008). PXR: A xenobiotic receptor of diverse function implicated in pharmacogenetics. Pharmacogenomics.

[69] Pascussi J.M., Robert A., Nguyen M., Walrant-Debray O., Garabedian M., Martin P., Pineau T., Saric J., Navarro F., Maurel P. (2005). Possible involvement of pregnane X receptor-enhanced CYP24 expression in drug-induced osteomalacia. J. Clin. Investig..

[70] Traber M.G. (2004). Vitamin E, nuclear receptors and xenobiotic metabolism. Arch. Biochem. Biophys..

[71] Landes N., Pfluger P., Kluth D., Birringer M., Ruhl R., Bol G.F., Glatt H., Brigelius-Flohe R. (2003). Vitamin E activates gene expression via the pregnane X receptor. Biochem. Pharmacol..

[72] Ruhl R. (2005). Induction of PXR-mediated metabolism by beta-carotene. Biochim. Biophys. Acta.

[73] Xie W., Tian Y. (2006). Xenobiotic receptor meets NF-kappaB, a collision in the small bowel. Cell Metab..

[74] Zhou C., Tabb M.M., Nelson E.L., Grun F., Verma S., Sadatrafiei A., Lin M., Mallick S., Forman B.M., Thummel K.E. (2006). Mutual repression between steroid and xenobiotic receptor and NF-kappaB signaling pathways links xenobiotic metabolism and inflammation. J. Clin. Investig..

[75] Pondugula S.R., Pavek P., Mani S. (2016). Pregnane X Receptor and Cancer: Context-Specificity is Key. Nucl. Recept. Res..

[76] Hakkola J., Rysa J., Hukkanen J. (2016). Regulation of hepatic energy metabolism by the nuclear receptor PXR. Biochim. Biophys. Acta.

[77] Oladimeji P.O., Chen T. (2018). PXR: More Than Just a Master Xenobiotic Receptor. Mol. Pharmacol..

[78] Rowland M.T.T. (2011). Clinical Pharmacokinetics: Concepts and Applications.

[79] Gesheff M.G., Franzese C.J., Bliden K.P., Contino C.J., Rafeedheen R., Tantry U.S., Gurbel P.A. (2014). Review of pharmacokinetic and pharmacodynamic modeling and safety of proton pump inhibitors and aspirin. Expert Rev. Clin. Pharmacol..

[80] De Jong H., Ropers D. (2006). Strategies for dealing with incomplete information in the modeling of molecular interaction networks. Brief. Bioinform..

[81] Ramakrishnan R., DuBois D.C., Almon R.R., Pyszczynski N.A., Jusko W.J. (2002). Fifth-generation model for corticosteroid pharmacodynamics: Application to steady-state receptor down-regulation and enzyme induction patterns during seven-day continuous infusion of methylprednisolone in rats. J. Pharmacokinet. Pharmacodyn..

[82] Ayyar V.S., Almon R.R., Jusko W.J., DuBois D.C. (2015). Quantitative tissue-specific dynamics of in vivo GILZ mRNA expression and regulation by endogenous and exogenous glucocorticoids. Physiol. Rep..

[83] Ayyar V.S., Sukumaran S., DuBois D.C., Almon R.R., Qu J., Jusko W.J. (2018). Receptor/gene/protein-mediated signaling connects methylprednisolone exposure to metabolic and immune-related pharmacodynamic actions in liver. J. Pharmacokinet. Pharmacodyn..

[84] Jiang X., Dutreix C., Jarugula V., Rebello S., Won C.S., Sun H. (2017). An Exposure-Response Modeling Approach to Examine the Relationship between Potency of CYP3A Inducer and Plasma 4beta-Hydroxycholesterol in Healthy Subjects. Clin. Pharmacol. Drug Dev..

[85] Leil T.A., Kasichayanula S., Boulton D.W., LaCreta F. (2014). Evaluation of 4beta-Hydroxycholesterol as a Clinical Biomarker of CYP3A4 Drug Interactions Using a Bayesian Mechanism-Based Pharmacometric Model. CPT Pharmacomet. Syst. Pharmacol..

[86] Goto A., Tagawa Y., Kimura Y., Kogame A., Moriya Y., Amano N. (2017). Influence of the pharmacokinetic profile on the plasma glucose lowering effect of the PPARgamma agonist pioglitazone in Wistar fatty rats. Biopharm. Drug Dispos..

[87] Hu L., Jin Y., Li Y.G., Borel A. (2015). Population pharmacokinetic/pharmacodynamic assessment of pharmacological effect of a selective estrogen receptor beta agonist on total testosterone in healthy men. Clin. Pharmacol. Drug Dev..

[88] Yamashita F., Sasa Y., Yoshida S., Hisaka A., Asai Y., Kitano H., Hashida M., Suzuki H. (2013). Modeling of rifampicin-induced CYP3A4 activation dynamics for the prediction of clinical drug-drug interactions from in vitro data. PLoS ONE.

[89] Kolodkin A., Sahin N., Phillips A., Hood S.R., Bruggeman F.J., Westerhoff H.V., Plant N. (2013). Optimization of stress response through the nuclear receptor-mediated cortisol signalling network. Nat. Commun..

[90] Luke N.S., DeVito M.J., Shah I., El-Masri H.A. (2010). Development of a quantitative model of pregnane X receptor (PXR) mediated xenobiotic metabolizing enzyme induction. Bull. Math. Biol..

[91] Bailey I., Gibson G.G., Plant K., Graham M., Plant N. (2011). A PXR-mediated negative feedback loop attenuates the expression of CYP3A in response to the PXR agonist pregnenalone-16alpha-carbonitrile. PLoS ONE.

[92] Lamba V., Panetta J.C., Strom S., Schuetz E.G. (2010). Genetic predictors of interindividual variability in hepatic CYP3A4 expression. J. Pharmacol. Exp. Ther..

[93] Slatter J.G., Templeton I.E., Castle J.C., Kulkarni A., Rushmore T.H., Richards K., He Y., Dai X., Cheng O.J., Caguyong M. (2006). Compendium of gene expression profiles comprising a baseline model of the human liver drug metabolism transcriptome. Xenobiotica.

[94] Kotta-Loizou I., Patsouris E., Theocharis S. (2013). Pregnane X receptor polymorphisms associated with human diseases. Expert Opin. Ther. Targets.

[95] Rana M., Devi S., Gourinath S., Goswami R., Tyagi R.K. (2016). A comprehensive analysis and functional characterization of naturally occurring non-synonymous variants of nuclear receptor PXR. Biochim. Biophys. Acta.

[96] Li L., Li Z.Q., Deng C.H., Ning M.R., Li H.Q., Bi S.S., Zhou T.Y., Lu W. (2012). A mechanism-based pharmacokinetic/pharmacodynamic model for CYP3A1/2 induction by dexamethasone in rats. Acta Pharmacol. Sin..

[97] Harley E.M., Loftus G.R. (2000). MATLAB and graphical user interfaces: Tools for experimental management. Behav. Res. Methods Instrum. Comput..

[98] Ghosh S., Matsuoka Y., Asai Y., Hsin K.Y., Kitano H. (2011). Software for systems biology: From tools to integrated platforms. Nat. Rev. Genet..

[99] Sheiner L.B., Grasela T.H. (1984). Experience with NONMEM: Analysis of routine phenytoin clinical pharmacokinetic data. Drug Metab. Rev..

[100] Pascussi J.M., Drocourt L., Fabre J.M., Maurel P., Vilarem M.J. (2000). Dexamethasone induces pregnane X receptor and retinoid X receptor-alpha expression in human hepatocytes: Synergistic increase of CYP3A4 induction by pregnane X receptor activators. Mol. Pharmacol..

[101] Oakley R.H., Cidlowski J.A. (1993). Homologous down regulation of the glucocorticoid receptor: The molecular machinery. Crit. Rev. Eukaryot. Gene Expr..

[102] Rigaud G., Roux J., Pictet R., Grange T. (1991). In vivo footprinting of rat TAT gene: Dynamic interplay between the glucocorticoid receptor and a liver-specific factor. Cell.

[103] Plant N. (2007). The human cytochrome P450 sub-family: Transcriptional regulation, inter-individual variation and interaction networks. Biochim. Biophys. Acta.

[104] Raybon J.J., Pray D., Morgan D.G., Zoeckler M., Zheng M., Sinz M., Kim S. (2011). Pharmacokinetic-pharmacodynamic modeling of rifampicin-mediated Cyp3a11 induction in steroid and xenobiotic X receptor humanized mice. J. Pharmacol. Exp. Ther..

[105] Gibson G.G., Phillips A., Aouabdi S., Plant K., Plant N. (2006). Transcriptional regulation of the human pregnane-X receptor. Drug Metab. Rev..

[106] Hargrove J.L. (1993). Microcomputer-assisted kinetic modeling of mammalian gene expression. FASEB J..

[107] Alberts B. (2008). Molecular Biology of the Cell.

[108] Claus J., Friedmann E., Klingmuller U., Rannacher R., Szekeres T. (2013). Spatial aspects in the SMAD signaling pathway. J. Math. Biol..

[109] Friedmann E. (2015). PDE/ODE modeling and simulation to determine the role of diffusion in long-term and -range cellular signaling. BMC Biophys..

[110] Thurley K., Gerecht D., Friedmann E., Hofer T. (2015). Three-Dimensional Gradients of Cytokine Signaling between T Cells. PLoS Comput. Biol..

